# A comprehensive evaluation of diversity measures for TCR repertoire profiling

**DOI:** 10.1186/s12915-025-02236-5

**Published:** 2025-05-14

**Authors:** Justyna Mika, Alicja Polanska, Kim RM Blenman, Lajos Pusztai, Joanna Polanska, Serge Candéias, Michal Marczyk

**Affiliations:** 1 Department of Data Science and Engineering, Silesian University of Technology, Gliwice, Poland; 2 Mullard Space Science Laboratory, University College London, Dorking, UK; 3 Yale Cancer Center, Yale School of Medicine, New Haven, CT USA; 4 Department of Computer Science, School of Engineering and Applied Science, New Haven, CT USA; 5 Université Grenoble Alpes, CEA, CNRS, IRIG-LCBM, Grenoble, France

**Keywords:** TCR profiling, Diversity, Richness, Evenness, TCR sequencing, Immunosequencing

## Abstract

**Background:**

T cells play a crucial role in adaptive immunity, as they monitor internal and external immunogenic signals through their specific receptors (TCRs). Using high-throughput sequencing, one can assess TCR repertoire in various clinical settings and describe it quantitatively by calculating a diversity index. Multiple diversity indices that capture the richness of TCRs and the evenness of their distribution have been proposed in the literature; however, there is no consensus on gold-standard measures and interpretation of each index is complex. Our goal was to examine the performance characteristics of 12 commonly used diversity indices in simulated and real-world data.

**Results:**

Simulated data were generated to evaluate how data richness and evenness affect index values using three nonparametric models. Fourteen real-world TCR datasets were obtained to examine differences in indices by analysis protocols and test their robustness to subsampling. *Pielou*, *Basharin*,* d50*, and *Gini* primarily describe evenness and highly correlate with one another. They are best suited for measuring the representation of TCR clones. Richness is best captured by *S* index, next *Chao1* and *ACE* which also consider information on evenness. *Shannon*, *Inv.Simspon*, *D3*, *D4*, and* Gini.Simpson* measure richness and increasingly more information on evenness. More skewed TCR distributions provided more stable results in subsampling. *Gini-Simpson*, *Pielou*, and *Basharin* were the most robust in both simulated and experimental data.

**Conclusions:**

Our results could guide investigators to select the best diversity index for their particular experimental question and draw attention to factors that can influence the accuracy and reproducibility of results.

**Supplementary Information:**

The online version contains supplementary material available at 10.1186/s12915-025-02236-5.

## Background

The latest high-throughput sequencing techniques allow the large-scale and rapid sequencing of B cell and T cell receptors (BCRs and TCRs) and quantification of clonotypes that are present in a tissue. Comparing TCR metrics between samples could inform about important immune characteristics of the host. Tools used in ecology to measure the biodiversity of an ecosystem are often applied to immunological sequencing data to measure the diversity of TCR and BCR sequences [1, 2]. The immune repertoire can be considered as a population of different clonotypes corresponding to species in ecology. The goal is to characterize the underlying species abundance distribution and compare these metrics across patient populations or assess association with disease outcome [3–12]. Choosing the best diversity measure to address a particular biological or clinical question is a non-trivial task and requires several interconnected factors to be considered. The question of how to quantify diversity has been the subject of debate in ecology for decades and has recently also gained interest in the context of immunology [13–17]. Most studies that examine immune repertoire diversities do not justify the choice of diversity metric and there is currently no consensus about gold-standard measures [15].

There is no universally accepted mathematical definition of diversity, but many heuristically defined diversity indices exist. A key and arguably the most intuitive characteristic of a species abundance distribution is the number of species in a population. It is measured by a straightforwardly defined index—the total number of species, known as richness. In immune repertoire data, a species can be understood as a unique TCR (or BCR) sequence. The second key characteristic is evenness, which measures how uniformly members of a population are divided among the various species present [18, 19]. Evenness is highest when all species have an equal number of members, and it is low when a small number of species contain most members of the population. In immune repertoire data, single copies of TCR (or BCR) sequences represent the population. A population that has a high richness and evenness is more diverse than a low-richness and low-evenness population. Various measures aim to capture both characteristics in a single diversity index. They vary in sensitivity and scaling behavior in response to changes in richness and evenness. There are also theoretical arguments to be made about the usefulness of various diversity measures [2, 20–22].

In real life setting, the data available for analysis represents only a sample taken from the entire population. This subsampling leads to what is called the unseen species problem in ecology. In the context of immune repertoires, experimental data often comes from a measurement of blood samples, or a tissue biopsy, that are unlikely to fully represent the entire immune repertoire of the host [14, 17]. Diversity measurements from a limited number of samples are influenced by sample size and also by experimental technical parameters. Diversity estimators are one approach to remedying the sampling problem. Estimators offer corrections for the number of unseen clonotypes. However, the assumptions required for these adjustments may not always be correct and more validation is needed to confirm the validity of these estimators [16, 17]. Comparison of different diversity metrics between sample sets is also challenging. When comparing samples using different diversity indices, one could observe opposing results. These may be due to a different relative importance of richness and evenness in the measures. Using diversity profiles has been suggested as a possible, but not well described solution [23, 24]. In diversity profiles, a range of values of a parameter controlling the measure’s sensitivity to rare species is considered, giving a more comprehensive picture. However, the lack of a single number representing the diversity can prove challenging when comparing large numbers of samples or using statistical tests. These methods also do not account for systematic measurement errors, which can differ between datasets, particularly if different measurement platforms are used.

In this article, we use computational methods to compare 12 commonly used diversity metrics in simulated and experimental TCR immune repertoire data. Our goal was to assess how data evenness, richness, and sequencing depth influence the performance of diversity measures. We also grouped the diversity indices based on shared performance characteristics. Our results could guide investigators in selecting the best method(s) for their particular experimental question and draw attention to factors that can influence the accuracy/reproducibility of results. Because of the significant overlap in the type of analytical questions, our results are also relevant to ecological studies of biodiversity.

## Results

### Diversity measures on simulated data

**Fig. 1:**
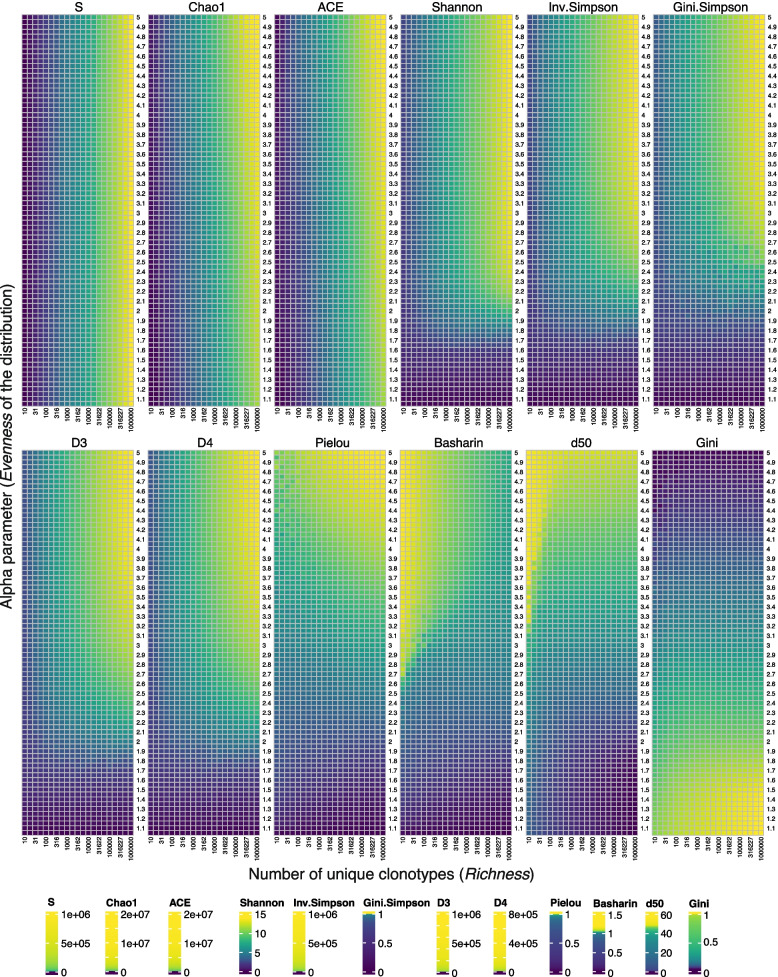
Heatmaps of mean diversity of 100 data simulation repeats for different Richness and Evenness parameters. The scale of colors is represented as quantiles of diversity index values. Diversity indices were grouped as described in the “Methods” section

To mimic real immunological datasets, we changed *Richness* from 10 to 1 million, and *Evenness* from 1.05 to 5.00. Small values of *Evenness* correspond to highly skewed TCR repertoires, and the distribution becomes uniform with increasing *Evenness* (11). We visualized the mean value of diversity indices calculated for every combination of *Richness* and *Evenness* (Fig. 1). Indices located in the upper left part (*S*, *Chao1*, and *ACE*) reflect mostly *Richness* and are insensitive to changes in *Evenness. Shannon*,* Inv.Simpson*,* Gini.Simpson*,* D3*, and *D4* indices consider both *Richness* and *Evenness* in changing ratios. They reflect changes in *Richness* for even distributions of TCRs and seem to be insensitive to such changes in the case of skewed TCR distributions (*Evenness* < 2). For the range of distributions from slightly skewed to slightly even, there is a nonlinear impact of *Richness* and *Evenness*. With increasing Hill number (*Shannon*: $q=1$; *Inv.Simpson* and *Gini.Simpson*: $q=2$; *D3*: $q=3$ and *D4*: $q=4;$ see Eq. 6), the threshold for distinguishing changes in only *Richness* increases, except for *Gini.Simpson*, whose response is visually more similar to Hill’s number $q=4$ than $q=2$. *Pielou*, *Basharin*, *d50*, and *Gini* mostly reflect changes in *Evenness*, with some nonlinearity caused by small *Richness* (*Richness* < 100). Gini has higher values for skewed distributions, which stays in opposite with the remaining indices in this group.

Next, we investigated how much variation is observed across values of diversity indices by calculating coefficients of variation (CV) for each value of *Richness* and *Evenness* separately (Additional file 1: Fig. S2). For different values in *Richness* (Additional file 1: Fig. S2 A)*,* no variation across *Evenness* was observed for *S* index, which is expected as values of *Richness* are equal to the values of *S*. Interestingly, *Chao1* and *ACE* show small variation across *Evenness* for all values in *Richness*. In the group of indices describing *Richness* and *Evenness*, the smallest variation across *Evenness* is observed for *Gini.Simpson*, and then *Shannon* indices. *Inv.Simpson*,* D3*, and *D4* have higher variation across *Evenness* for increasing *Richness*. In the group of indices describing just *Evenness*,* Pielou*, and *Basharin* have small variations across *Evenness* over all values of *Richness*. Next, for different values in *Evenness*, we checked how much variation across *Richness* is presented by all the indices (Additional file 1: Fig. S2B). Here, indices designed to describe just *Richness (S, Chao1,* and *ACE)* had the highest and most stable variation across *Richness* for all values of *Evenness*. In the group of indices describing *Richness* and *Evenness*, almost no variation is observed for *Gini.Simspon* for all *Evenness* values. Small variation across *Richness* is observed for *Shannon* index and increasing variation with increasing *Evenness* for *Inv.Simpson*,* D3*, and *D4*. Interestingly for very skewed distributions (*Evenness* < 1.6), there is almost no variation across *Richness* for all indices in this group. Finally, in the group of indices designed to describe just *Evenness*, there is almost no variation across *Richness* for *Gini* index. *Pielou* and *Basharin* have some variation across Richness for very skewed distributions, whereas *d50* has a very high variation for skewed distributions. In this group of indices, there is no variation across *Richness* for highly even distributions (*Evenness* > 4).

**Fig. 2:**
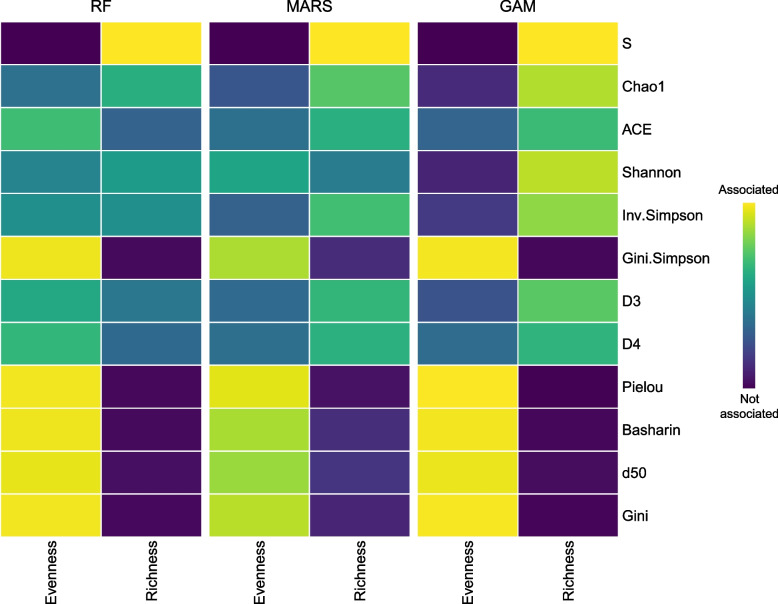
Evaluation of how different diversity metrics reflect changes in Richness and Evenness by variable importance. Variable importance was calculated from three nonparametric models: random forests (RF), multivariate adaptive regression spline (MARS), and generalized additive model (GAM). Euclidean distance was used for clustering the diversity methods regarding the GAM model

Finally, to quantify how diversity measures reflect the *Evenness* and *Richness* of the TCR repertoires, we fitted three non-parametric models (RF, GAM, and MARS) and calculated the importance of these two variables for every diversity index (Fig. 2). All created models were statistically significant with a high coefficient of determination (R^2^). While the R^2^ coefficient was very high for most of the indices (R^2^ > 0.7), it was visibly smaller (in the range of 0.40–0.55) for MARS and GAM model for *Inv.Simpson*, *D3*, *D4*, and *ACE*. The results of variable importance analysis show that the group of indices categorized as describing only *Evenness*, i.e., *Pielou*,* Basharin*,* d50*, and *Gini*, indeed almost entirely describe *Evenness*. Interestingly, *Gini.Simspon* seems to also fit this group very well. These indices can be utilized for the analysis of the representation of TCR clones in the population, independent of the number of unique TCR clones in the population. *Richness* is best reflected by *S* index, meaning that it can be used for the analysis of the number of unique TCR clones in a population regardless of their distribution. *Chao1* and *ACE* seem to fit better the group of indices, sensitive to both *Richness* and *Evenness*, even though they show more association with *Richness*. For the remaining indices, the impact of *Richness* diminishes with increasing Hill numbers (corresponding to the following order of indices: *Shannon*, *Inv.Simpson*, *D3*, and *D4*). Thus, the higher the Hill number (higher *q* in Eq. 6), the more impact is put on the distribution of TCR clones in a population and less on the number of unique TCR clones.

### Diversity measures on real data

**Fig. 3:**
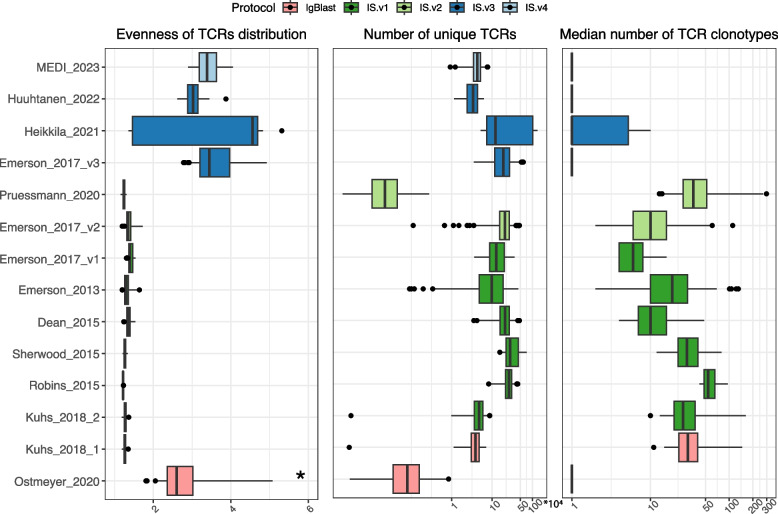
Basic characteristics of the TCR repertoires in real-world datasets. The Evenness of the distribution was calculated as an alpha parameter estimated with power law distribution (see Eq. 1). A star (*) corresponds to an extremely outlying sample in Ostmeyer_2020 set (Evenness = 30). The number of unique TCRs is shown in 10,000 s on the *x*-axis of the middle panel. Protocols of the datasets correspond to the annotation method applied: IgBlast = IgBlast and IS = ImmunoSeq (see “Methods” for further details). IS.vx corresponds to the counting method applied within the ImmunoSeq protocol

Next, we used the collection of experimental datasets with TCR repertoires (Table 1) to investigate the robustness of diversity indices. We calculated the basic characteristics of the functional part of TCR repertoires in all analyzed datasets (Fig. 3). Most of the datasets were annotated with ImmunoSeq (IS.vx), but using different counting methods (v1, v2, v3, and v4). Older counting methods (v1 and v2), which used reads in calculating the frequency of a rearrangement, resulted in highly skewed TCR distributions with a high number of unique TCRs and a high median number of unique TCR copies (clonotypes). The newest counting methods (v3 and v4), which use templates (i.e., number of biological molecules applied for sequencing) for quantifying rearrangements, resulted in rather uniform TCR distributions with a high number of unique TCRs and a very small number of TCR clonotypes (median number of TCR clones = 1). The observed difference between counting methods is obvious when comparing samples from the same dataset (Emerson_2017; v1 *vs* v2 *vs* v3). Two datasets obtained with IgBlast represent one set of skewed and one set of uniform distributions of TCR clones.

**Table 1:** Description of analyzed real-world datasets

	**Dataset**	**Annotation tool**	**Version**	**Sample type**	**Biological condition**	***N***	**Outliers**
1	MEDI_2022 [25]	ImmunoSEQ	v4	Blood	Breast cancer chemotherapy response (pCR versus RD)	48	1
2	Huuhtanen_2022 [26]	ImmunoSEQ	v3	Blood	Prior Treatment vs No Prior Treatment	21	0
3	Heikkila_2021 [27]	ImmunoSEQ	v3	Blood and thymus tissue	Blood vs Thymus	12	0
4	Emerson_2017_v3[28]	ImmunoSEQ	v3	Blood	CMV + vs CMV −	119	0
5	Pruessmann_2020 [29]	ImmunoSEQ	v2	FFPE tissue blocks	Male vs Female	199	15
6	Emerson_2017_v2[28]	ImmunoSEQ	v2	Blood	CMV + vs CMV −	567	1
7	Emerson_2017_v1[28]	ImmunoSEQ	v1	Blood	CMV + vs CMV −	74	0
8	Emerson_2013 [30]	ImmunoSEQ	v1	Blood	CD4 vs CD8	118	3
9	Dean_2015 [31]	ImmunoSEQ	v1	Blood	Male vs Female	564	4
10	Sherwood_2015 [32]	ImmunoSEQ	v1	Blood	-	39	0
11	Robins_2015 [33]	ImmunoSEQ	v1	Blood	Male vs Female	26	1
12	Kuhs_2018_2 [34]	ImmunoSEQ	v1	Blood	HPV infection vs no HPV infection	60	2
13	Kuhs_2018_1 [34]	IgBlast	-	Blood	HPV infection vs no HPV infection	60	2
14	Ostmeyer_2020 [35]	IgBlast	-	FFPE tissue blocks	-	60	0

The biological condition column shows the factor for which statistical testing was performed. *N* corresponds to the number of samples in a dataset with complete information about biological conditions (if available). For Ostmeyer_2020, there is no clinical data provided. Sherwood_2015 is not a case–control study, thus no statistical testing was performed.

**Fig. 4:**
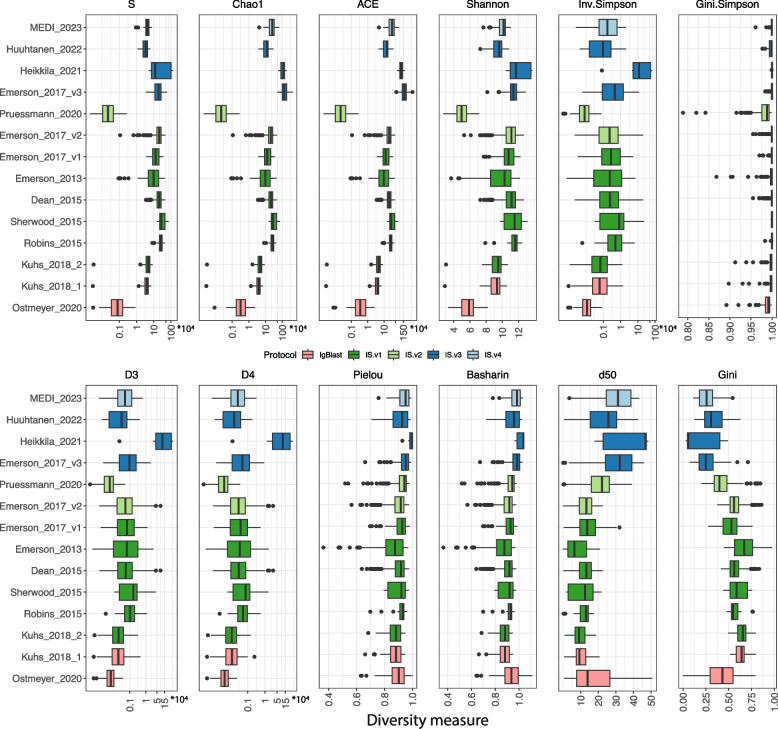
Distribution of calculated diversity indices for every dataset. A dot represents an outlying repertoire based on Tukey mild outliers’ detection method. S, Chao1, ACE, Inv.Simpson, D3, and D4 indices are shown in logarithmic scale

We calculated diversity indices for every unique repertoire in all datasets (Fig. 4). The highest variation per dataset is for the Heikkila dataset, which consists of only 12 repertoires with highly varied richness and evenness of the TCR distributions, most probably due to the different origins of samples (blood and thymus tissue). The smallest variation of diversity indices is observed for the Robins_2015 dataset, which consists of repertoires of high skewness of TCR distribution and a high number of unique TCR sequences. The spread of values that a unique diversity index can get is very wide. The highest variation of diversity indices is observed for *ACE*,* Chao1*,* S*,* Inv.Simpson*,* D3*, and *D4* measures. The smallest variation is observed for *Gini.Simpson*,* Pielou*, and *Basharin*. All get values close to 1, resulting in highly skewed distributions of index values. Kuhs_2018_1 and Kuhs_2018_2 datasets, which are the same data but annotated with two different protocols, resulted in almost the same values of diversity indices in both cases. For Emerson_2017, where numbers of TCR copies were estimated using three different counting methods, for most of the indices, the range of obtained values is the biggest for v2 with the highest number of outliers; however, the interquartile range is smaller or equal to the ones from v1 and v3 methods. For the indices designed to describe only richness or evenness, samples analyzed with v3 method have higher values than the remaining samples (except for *Gini* index, for which we observe an opposite relationship). This impact may be caused either by the counting method or by different characteristics of donors examined in particular batches of Emerson_2017 dataset. There seems to be no difference in the spread of indices designed to describe both evenness and richness between the counting methods.

**Fig. 5:**
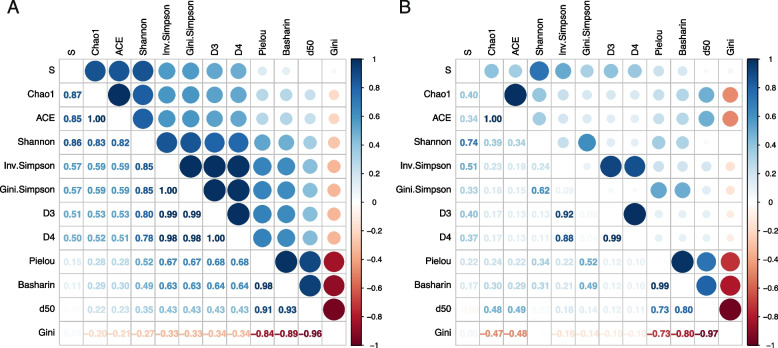
Spearman (**A**) and Pearson (**B**) correlation coefficients between diversity indices across all datasets.Positive correlation is colored blue and negative correlation is shown in the shades of red. The size of each dot corresponds to the strength of correlation—the bigger the dot, the stronger the association

To investigate if the designed groups of indices hold on in real-world datasets, we calculated pairwise correlations between all diversity indices, considering diversity results for all repertoires in all datasets at the same time (Fig. 5). Based on the Spearman correlation, which measures a monotonical association between the diversity indices (Fig. 5A), we observe strong correlations between indices in all three groups of indices: describing only richness, describing only evenness, and describing both. Interestingly, indices for richness description (*S*,* Chao1*, and *ACE*) strongly correlate with indices describing both evenness and richness (*Shannon*,* Inv.Simpson*,* Gini.Simpson*,* D3*, and *D4*) and show small or negligible correlation with indices describing just evenness (*Pielou*,* Basharin*,* d50*, and *Gini*). There is a medium to a high correlation between groups of indices designed to describe both evenness and richness and those describing only evenness in the following order: *Gini*,* d50*,* Basharin,* and *Pielou*. Next, we checked the linear relationship between the indices using the Pearson correlation (Fig. 5B). Here we observe a very high relationship between the indices describing only evenness (*Pielou*,* Basharin*,* d50*, and *Gini*). In the group of indices describing only richness, there is an almost ideal correlation between *Chao1* and *ACE*; however, *S* is associated with them at a medium level. In the group of indices describing both richness and evenness, there is a very high correlation between *Inv.Simpson*,* D3*, and *D4*. *Gini.Simspon* is linearly correlated with *Shannon* index and interestingly, also with *Pielou* and *Basharin. S* is also correlated with indices describing both richness and evenness, whereas *ACE* and *Chao1* show small to medium correlation with all indices for evenness description and *Shannon* index.

Prompted by these findings, we estimated how various diversity indices differ between TCR repertoires from two distinct biological conditions in each dataset (as indicated in Table 1). We calculated the rank biserial correlation to quantify effect size differences between indices when comparing two biological groups (Table 2). The effect size (i.e., absolute difference in index value between the two comparison groups) was high or very high in Emerson_2013 for all analyzed indices. Moreover, the observed difference between the biological conditions was also statistically significant. Emerson_2017 (v1, v2, and v3 datasets) show significant differences between the analyzed biological conditions for all indices describing only evenness and both evenness and richness. Worth noticing that v2 dataset had the smallest effect size value when compared to v1 and v3. For Heikkila_2021, the effect size was very high for indices describing only richness and both richness and evenness, which might reflect significant differences between TCR repertoires obtained from blood and directly from thymus. It must be, however, noticed that this dataset consisted of only 12 samples. The smallest variations of results are observed for Pruessmann_2020, MEDI_2023, and Robins_2015 datasets; however, in all these datasets there is also no difference between the biological conditions analyzed in this study. Indices categorized as the ones describing only richness have small or no variation of values for all the datasets (standard deviations of effect size measures smaller than 0.09), where no variation is observed for skewed distributions.

**Table 2:** Effect size (RBCC) of a difference between case–control groups. The closer the RBCC is to 1, the bigger the difference between the groups. Statistically significant comparisons (*p*-val < 0.05) are shown in bold and italics. *Sherwood_2015 is not a case–control study and for Ostmeyer_2020 there is no clinical data provided, thus no statistical testing was performed in these two cases

Method	S	Chao1	ACE	Shannon	Inv Simpson	Gini Simpson	D3	D4	Pielou	Basharin	d50	Gini
MEDI 2023 [25]	0.072	0.014	0.000	0.083	0.033	0.033	0.047	0.047	0.007	0.000	0.014	0.007
Huuhtanen 2022 [26]	0.206	0.029	0.147	0.265	0.618	0.618	0.647	***0.676***	0.441	0.441	0.294	0.324
Heikkila 2021 [27]	***1.000***	***1.000***	***0.875***	***1.000***	***1.000***	***1.000***	***1.000***	***1.000***	0.250	0.313	0.063	0.063
Emerson 2017_v3 [28]	0.092	0.016	0.021	***0.424***	***0.602***	***0.602***	***0.603***	***0.596***	***0.502***	***0.482***	***0.307***	***0.319***
Pruessmann 2020 [29]	0.043	0.043	0.043	0.055	0.027	0.027	0.023	0.011	0.027	0.023	0.022	0.032
Emerson 2017_v2 [28]	0.025	0.025	0.025	***0.159***	***0.212***	***0.212***	***0.198***	***0.192***	***0.219***	***0.216***	***0.181***	***0.171***
Emerson 2017_v1 [28]	0.245	0.245	0.245	***0.526***	***0.550***	***0.550***	***0.528***	***0.512***	***0.539***	***0.531***	***0.443***	***0.340***
Emerson 2013 [30]	***0.565***	***0.565***	***0.565***	***0.756***	***0.867***	***0.867***	***0.877***	***0.874***	***0.752***	***0.749***	***0.579***	***0.553***
Dean 2015 [31]	***0.144***	***0.144***	***0.144***	***0.138***	0.082	0.082	0.074	0.072	0.092	0.092	0.088	0.089
Robins 2015 [33]	0.074	0.074	0.074	0.015	0.088	0.088	0.191	0.250	0.088	0.088	0.103	0.176
Kuhs 2018_2 [34]	***0.520***	***0.520***	***0.520***	0.291	0.094	0.094	0.077	0.073	0.103	0.103	0.089	0.170
Kuhs 2018_1 [34]	***0.551***	***0.551***	***0.551***	0.251	0.025	0.025	0.027	0.020	0.068	0.068	0.056	0.146

Finally, we investigated the robustness of diversity indices for varying sequencing depth, equivalent to the total number of TCR sequences. For every dataset, we subsampled TCR sequences, controlling the total number of sequences. We tested a wide range of subsampled sequences, starting from the minimal number of total TCR sequences detected in all datasets (i.e., 36 sequences in Ostmeyer_2020 dataset) up to the median number of TCR sequences in each dataset. We repeated random subsampling 100 times for each sequencing depth and calculated all diversity indices (Additional file 1: Fig. S3). To measure the robustness of indices, we calculated coefficients of variation (CV) and relative error (RE) in repeated subsampling. CV close to zero represents a robust index, having a stable value for all 100 repeats of subsampling, whereas RE close to zero indicates a non-biased index giving a similar value to primary non-sampled data. Next, to investigate the impact of sequencing depth on the robustness of indices, we calculated the median values of CV and RE for every diversity index and dataset (Additional file 1: Fig. S4). To inspect if there is a change of CV and RE with sequencing depth, we fitted a linear regression model to get the slope coefficient. A slope coefficient closer to 0 means a more stable index across the sequencing depth (Additional file 1: Fig. S5).

**Fig. 6:**
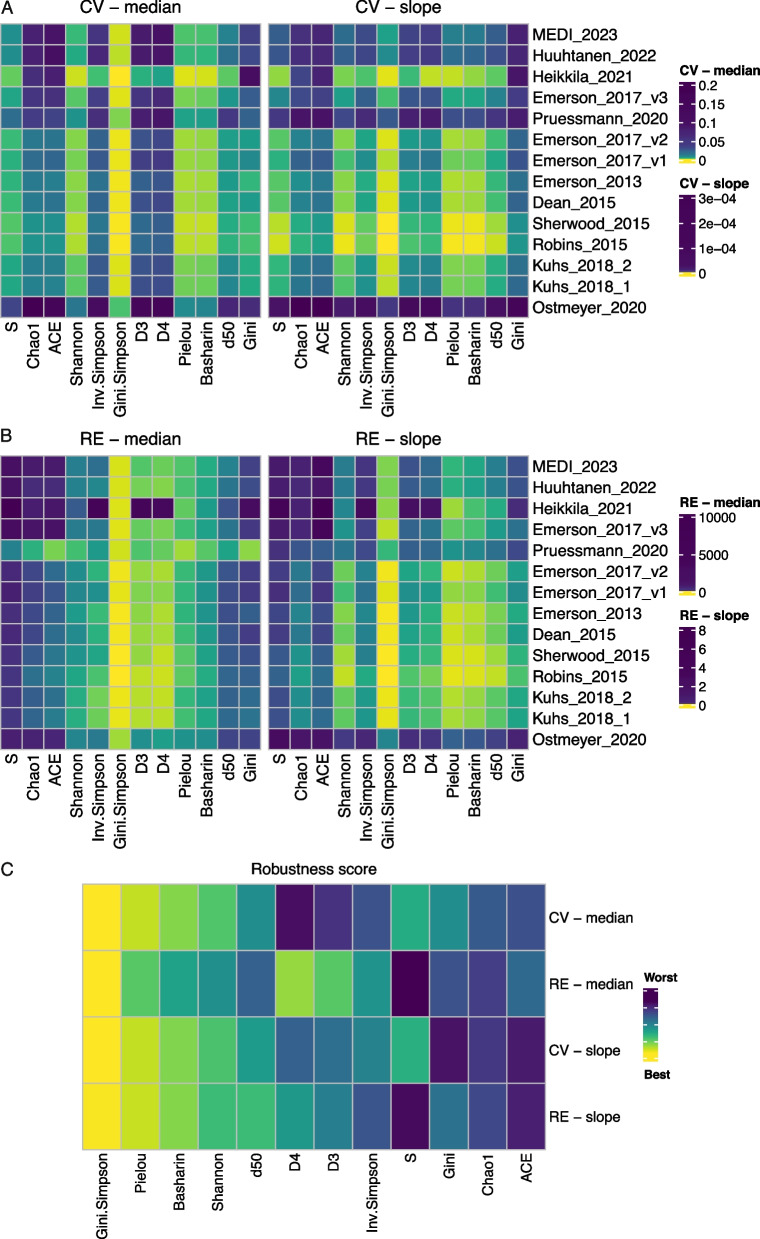
Robustness of diversity indices to data subsampling. For coefficient of variation (CV) and relative error (RE), median values are shown together with a slope coefficient from linear models of CV and RE. Values closer to 0 (colored yellow) represent a more robust index across varying sequencing depths. Panel **A** shows results for CV, panel **B** shows results for RE, and panel **C** shows the summary ranking of methods regarding their robustness score

**Fig. 7:**
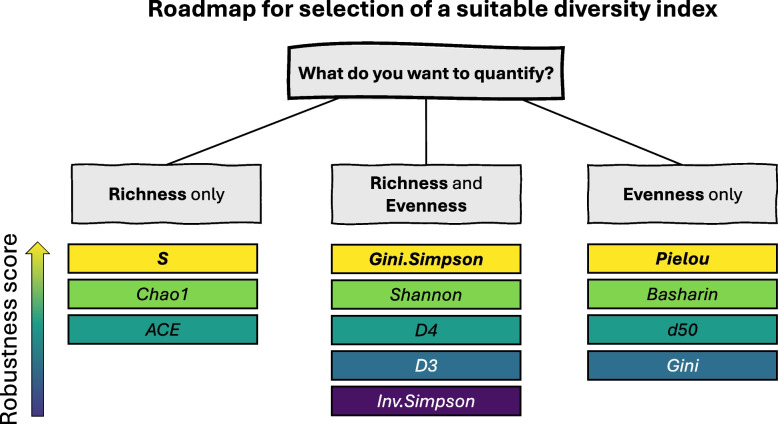
Roadmap for selection of the most robust diversity index under different experimental questions

Skewed TCR distributions have in general lower median values of CV as well as CV slope coefficients, than the uniform TCR distributions (Fig. 6A). The exceptions are presented by Heikkila_2021 dataset, which even though has a uniform TCR distribution, shows lower values of CV, and by Pruessmann_2020 dataset, which even though has skewed distributions, its CV values are quite high. *Gini.Simpson* index has the lowest value of median CV in all datasets and its values were rather stable across the increasing sequencing depths. Next in line are *Pielou*,* Basharin*, and *Shannon* indices, which had low median CV and slope coefficients of the model. In the group of indices describing richness, *S* seems to be more robust than *Chao1* and *ACE*. Looking at the robustness of indices regarding the differences from non-sampled value (Fig. 6B), similarly as for the CV results, skewed distributions in general show lower variation and stable response for the increasing sequencing depth regarding the relative error. The *Gini.Simpson* index again shows the lowest median RE and slope of RE, meaning that the results after subsampling are very close to the original value on a non-sampled dataset. Hill numbers *D3* and *D4* have also a small median value of RE, which contrasts with the high median value of the CV. This shows that indices designed to describe both richness and evenness have the lowest values of relative error. The impact of increasing sequencing depth on RE changes is similar to the results for CV. Here, apart from *Gini.Simpson*, the most robust indices are the ones describing only evenness (*Pielou, Basharin,* and *d50)*. Moreover, *S* shows high differences between subsampled and original results, which stays in contrast to CV results. To summarize the results of the diversity index robustness analysis, we constructed a ranking of methods and calculated the robustness score (Fig. 6C). The top three robust indices are *Gini.Simpson*,* Pielou*, and *Basharin*, which result in the lowest values of median CV and slope of CV and RE. Based on these results, we constructed a roadmap for the selection of the most robust diversity index under different experimental questions (Fig. 7).

## Discussion

Comparing distinct TCR repertoires is not straightforward since there are multiple indices that measure diversity [14]; however, there is currently no consensus about the gold-standard best measure [15]. Moreover, to the best of our knowledge, there has not yet been a study performing a comprehensive comparison and evaluation of the factors that influence the performance of different diversity indices and assess their robustness. Chiffelle et al. checked variations of *S*, *Gini*, *Gini.Simpson*, and *Pielou* upon different values of *Richness* and *Evenness*, however only within a limited range of unique and total TCR counts, and on simulated data only [15]. Roswell et al. evaluated *S*, *Shannon*, and *Simpson* on ecological data [36]. Studies performing TCR repertoire analysis typically choose one or few of the many diversity indices, making a comparison of different studies [15] and meta-analysis impossible. Also, immunosequencing experiments typically yield wide ranges of coverage, equivalent to wide ranges of total numbers of TCR sequences. These large variations in TCR counts in different studies make it challenging to propose an index resulting in robust results regardless of the experiment’s coverage. Finally, TCR repertoires can be described by the richness (equivalent to the number of unique TCR sequences) and evenness of the TCR distribution (informing about the frequency of the unique TCR clones), and different metrics may need to be used to robustly measure these different features of the data. In this study, we performed a comprehensive assessment of the sensitivity of 12 different diversity metrics to varying *Evenness* and *Richness* of simulated datasets and examined the robustness of results in subsampling in experimental TCR repertoire datasets.

We categorized the indices into three groups: (i) describing only richness of the distributions (*S*,* Chao1*, and *ACE*); (ii) describing both richness and evenness (*Shannon*,* Inv.Simpson*,* Gini.Simpson*,* D3*, and *D4*); and (iii) describing only evenness of the distribution (*Pielou*,* Basharin*,* d50*, and *Gini*). Our findings confirm such division, with few interesting exceptions. In the group of richness describing indices, *S* is equivalent to the number of unique TCR sequences, so it resembles richness perfectly. *Chao1* and *ACE* on the other hand mostly describe richness, however, also consider information on evenness (Figs. 1 and 2). The following indices consider both richness and increasingly more information about the evenness of TCR sequences: *Shannon, Inv.Simpson*,* D3*,* D4.* We note that the order of these indices corresponds to the increasing Hill numbers (*Shannon* is equivalent to D1, and *Inv.Simpson* to D2), which consider information about the relative abundance of more frequent clones. For these indices, the importance of the evenness and richness is different depending on the model; however, the trend of increasing importance of evenness with increasing Hill number is stable for all models applied (Fig. 2). Thus, the higher the Hill number, the more weight is put on the distribution of TCR clones in a population and less on the number of unique TCR sequences in a repertoire. By analyzing simulation data results, *Gini.Simpson*, originally assigned to the group of indices describing both richness and evenness, seems to fit more into the group of indices describing mostly evenness (Fig. 2). Also, the pattern of its changes regarding changing *Evenness* and *Richness* (Fig. 1) resembles more the results for the high Hill number (*D4*), than the actual D2 it is. The indices describing almost entirely evenness of the distribution are *Pielou*,* Gini.Simpson*,* Gini*,* Basharin*, and *d50* (Figs. 1 and 2). *Richness* does not impact the values of these indices (Fig. 2), and the results are highly correlated and consistent between different nonparametric models. These indices can be utilized in the analysis focusing on the representation of TCR clones in a population, independent of the number of unique TCR sequences in a repertoire.

The above grouping of indices is further validated by the correlation analysis performed on real data (Fig. 5). Here, however, *Gini.Simpson* values much stronger correlate monotonically with indices describing both the evenness and richness of the distribution, rather than with evenness-only indices. Looking at the linear association between the indices, the *Gini.Simpson* index is only correlated with *Shannon*, *Pielou*, and *Basharin* (*r* = 0.62, 0.52, and 0.49 respectively), which indicates that its distribution is very dissimilar to others (highly skewed). Linear association of the indices allows us to distinguish more specific groups of indices which are almost entirely correlated with each other. Even though their values might be different for a given repertoire, they will change proportionally for the changing richness/evenness of the repertoire. *Chao1* is almost entirely correlated with *ACE* (*r* = 1.00). The next group consists of *Inv.Simpson*, *D3*, and *D4* indices (*r* = 0.88–0.99). *Pielou* is almost entirely correlated with *Basharin* (*r* = 0.99), whereas *d50* is negatively correlated with *Gini* (*r* = − 0.97). Finally, the whole group of evenness describing indices is well correlated with each other (*Pielou*,* Basharin*,* d50*, and *Gini*; *r* in the range of 0.73–0.99). This suggests that such groups of indices provide similar interpretations of TCR population diversity and can be used to answer the same scientific questions. One must however remember that *Gini* index results are in a negative association with all the remaining indices.

Simulated data were generated using a discrete power law distribution, controlling the *Richness* and *Evenness* of the TCR distribution [37]. Hence, the resulting dataset contained information on the underlying richness and evenness of the repertoires that was leveraged to gain a deeper understanding of the meaning of individual metrics. The generated repertoires resulted in a wide range of total counts of TCR sequences. All the indices, except for the *Basharin* estimator of entropy, are blind to the total number of TCR sequences (coverage), considering only the relative abundances of clones. When calculating the *Basharin* index, information about the total number of TCR sequences is used for the entropy estimation, which causes a bias for specific repertoires. This bias is especially observed for the repertoires of even distributions with low richness (Fig. 1), represented mostly by repertoires of singular TCR clones. In such repertoires, unique and total counts have similar values, resulting in an excessive increase of the correction part within the *Basharin* index. Thus, the *Basharin* index is not recommended for the analysis of repertoires with rather uniformly distributed TCR clones. It is worth noting that experimental TCR sequencing data usually result in high unique and total counts of TCR sequences (here median number of unique and total counts across all datasets equals 127,736 and 2,757,364 respectively).

One of the objectives of many research studies is a comparative analysis of biological conditions between sets of samples. Here, we performed a nonparametric test with corresponding effect size statistics to measure the differences in TCR repertoires between study-specific biological groups. High effect size and statistically significant differences were observed mostly either for the group of indices describing only richness or for the group of all remaining indices, depending on the dataset. The exception here is Heikkila_2021 dataset, for which the highest effect size (RBCC = 1) was observed for indices describing only richness and those describing both richness and evenness. Interestingly, indices categorized as the ones describing only richness have no variation of effect size values for skewed distributions and a small variation of effect size values for the uniform distributions (standard deviation < 0.09). Emerson_2013 dataset was characterized by the skewed distribution of TCRs and resulted in significant differences between the CD4^+^ and CD8^+^ cell subsets under comparison. Emerson et al. showed significant differences in V gene usage between CD4^+^ and CD8^+^ cells [30], also the differences between the CD4^+^ and CD8^+^ cell subsets evenness changes in age were observed [38, 39]. For this dataset, high and significant effect size statistics were measured for all diversity indices, where the highest difference was observed for indices measuring both richness and evenness. For Emerson_2017 dataset, statistical differences between CMV − and CMV + patients were observed for evenness-only indices and also the one estimating both richness and evenness. Worth noticing, even though this dataset presents subsets of skewed (v1 and v2) and uniform (v3) distributions of repertoires, a significant difference is observed in all. Interestingly, v2 subset results in small differences between biological conditions, whereas v1 and v3 correspond to high or medium effect size. This might be explained by the different spreads of diversity index values obtained by these methods (Fig. 4), where diversity indices for v2 dataset had usually a narrower interquartile range with the biggest number of outliers.

The skewness of the distribution strongly impacts the robustness of diversity indices, as indicated by the subsampling experiments, as well as by variations observed in simulated data. For each metric, there is a clear *Evenness* threshold (parametrized by the alpha parameter of the power law distribution), which distinguishes the high and low variation of diversity measurement (Additional file 1: Fig. S2B). *Shannon*, *Inv.Simpson*,* D3*, and *D4* have high variation for uniform distributions and show almost no variation for skewed TCR distributions (*Evenness* < *2)*. On the opposite side are *d50*, *Pielou*, and *Basharin*, which show higher variation for skewed distributions. Moreover, the subsampling results indicate that skewed TCR distributions result in more robust diversity measurements compared to more uniform distributions (Fig. 6). This is especially of great importance, as we found a high bias of rearrangement counting methods on the distribution of TCR clones (Fig. 3). Previous counting methods resulted in skewed distributions of TCRs, whereas new counting methods provide more uniform ones. Thus, it might be argued that the skewness of the distribution is highly dependent on the counting method used.

The majority of datasets analyzed in this study originated from blood samples, except for two datasets: Pruessman_2020 and Ostmeyer_2020, which were obtained from FFPE tissue blocks. It is well known that nucleic acids get degraded during storage in formalin-fixed and paraffin-embedded samples, which impacts the robustness of sequencing methods [40–42]. In our analysis, Pruessman_2020 samples were measured by immunoSEQ assay and resulted in skewed distributions, whereas Ostmeyer_2020 used IgBlast to estimate the numbers of TCR rearrangements and resulted in rather uniform TCR distributions. Despite differences in counting methods used, both datasets resulted in the smallest number of unique TCR clones (equivalent to richness) among all datasets (Fig. 3). The impact of small richness is also observed on diversity indices values, not only on those measuring richness but also on indices measuring both richness and evenness. In all these cases, Pruessman_2020 and Ostmeyer_2020 samples obtained the smallest values (Fig. 4). Finally, the robustness of different diversity indices on sequencing depth was in most cases the smallest for these two datasets (Fig. 6A, B). This might be caused by a very low number of TCR sequences in the original samples, causing the range of subsampling to be rather narrow. It must be however noted that the relative to the other indices changes of coefficient of variation were in pair with the remaining datasets, whereas the analysis of relative error showed quite different interpretations, especially for Pruessmann_2020 dataset. Altogether, these results confirm the adverse effect of FFPE storage on sequencing quality, indicating that one should be careful while analyzing these data.

Most of the analyses showed that among the twelve tested diversity measures, the *Gini.Simpson* index provides the most robust diversity measure for both simulated and experimental data. It has the lowest variation across different *Richness* and *Evenness* values of the distribution and displays the most stability under subsampling. Values of *Gini.Simpson* theoretically range from 0 to 1; however for analyzed real and simulated data, it is typically higher than 0.8, independent of the evenness of the distribution (Fig. 1 and Fig. 4). Even though the spread of the *Gini.Simpson* values was very narrow, the statistical testing results were on par with the other indices. Next in line indices showing the most robust results are *Pielou* and *Basharin*, which are associated mostly with the description of TCR repertoire distribution. These indices also get values from quite narrow range (0–1 for *Pielou* and 0–1.2 for *Basharin*). For the problem of estimating a unique number of TCR clones in a repertoire, *S* index seems to be the most stable and robust one. To summarize these results, we propose the following indices in particular case of TCR repertoire analysis: (i) *S* to quantify richness; (ii) *Gini.Simpson* to quantify both richness and evenness; and (iii) *Pielou* to quantify evenness (Fig. 7).

Even though we observed meaningful results on the robustness of a wide range of diversity measures, there are some limitations of this study. First, during the generation of simulated datasets, we did not model any noise observed in real data, arising from technical issues like library preparation or signal amplification. Such noise might also bias the results of specific indices and their robustness, but the noise models for this type of data are not described yet. Moreover, we utilized experimental data obtained only with two different sequencing protocols, whereas there are many more available (ex. MiXCR [43], TRUST4 [44], ImReP [45]), although less commonly used in the literature. In future studies, it could be informative to consider the impact of more parameters on diversity metrics, like different platforms for TCR repertoire measurements, including RNA-based assays; the experimental parameters like amount of input DNA, or different loci of T cell rearrangements (i.e., alpha or gamma chains). Finally, we acknowledge that there are other diversity measures proposed in literature beyond the twelve that we considered in this work. We chose to restrict the scope of our analysis to the most frequently used methods and metrics that capture various aspects of the distribution.

## Conclusions

We performed a comprehensive evaluation of twelve commonly used diversity measures for TCR profiling on simulated and experimental datasets. We categorized indices into groups depending on their ability to describe only richness of the distribution, only evenness of the distribution, or both. Using simulated data, we verified that indices in particular groups are categorized properly. We showed that there is a high impact of skewness of distribution on the robustness of diversity indices; skewed distributions tend to provide more stable results. This conclusion is of high importance, given the high impact of sequencing protocol on the skewness of measured repertoires. Among all tested indices, *Gini.Simpson*, *Pielou*, and *Basharin* resulted in the most robust outcomes for both simulated and experimental data.

## Methods

### Data simulation

To systematically study the impact of different degrees of sample richness and evenness on the various diversity indices, clonotype abundance data were simulated. We created simulated data with wide ranges of numbers of unique TCR sequences (*Richness*) and varying skewness of the TCR distribution (*Evenness*). Counts of different clonotypes were treated as a discrete random variable *X* generated from a power law distribution using the R-language package *poweRlaw* and *rpldis* function [37]. For each sample, a set of number of counts, *S*, was generated according to the probability mass function [37], with the lower bound equal to $1$, which is the smallest possible non-zero clonotype count:

1 $$P(X=x)=\frac{{x}^{-\alpha }}{\zeta (\alpha )}$$

where $\alpha>1$ is a real parameter that controls the evenness of the distribution and $\zeta (\alpha )$ is the normalizing constant given by the Riemann zeta function. Count data were generated for $\alpha$, denoted as *Evenness*, in a range between $1.05$ and $5$ in increments of $0.05$, and for $S$, denoted as *Richness*, in a range between ${10}^{1}$ and ${10}^{6}$ changing the exponent in increments of $0.25$ and rounding non-integer numbers down. For each unique combination of *Evenness* and *Richness*, the data was generated at random $100$ times.

### Real data

All TCR repertoires used in this study were downloaded from either ImmuneAccess [37, 37] (https://clients.adaptivebiotech.com/immuneaccess) or VDJServer [46] platforms (Table 1). In all cases, genomic DNA samples were sent to Adaptive Biotechnologies [38, 38] (https://www.adaptivebiotech.com/adaptive-immunosequencing) for sequencing of the TCR β locus, using immunoSEQ protocol and sequenced with Illumina platform. The data were further processed with the immunoSEQ annotation tool, except for Kuhs_2018_1 and Ostmeyer_2020 datasets, for which IgBlast was applied. There are subsequent versions of immunoSEQ Assay annotation tools, which use either reads (v1, v2) or templates (v3, v4) to estimate the number of copies of a unique TCR in a repertoire. Based on the Adaptive Biotechnology company technical report, a read is defined as a sequenced copy of a DNA template, whereas templates refer to the number of biological molecules put into the assay before PCR amplification and sequencing.

### Data preprocessing

Nonfunctional sequences, i.e., sequences out of frame or containing a stop codon, were filtered out from each sample. The evenness of the TCR distribution was estimated based on a power law distribution (Eq. 1) [37]. The $\alpha$ parameter that describes the TCR distribution was found by a maximum likelihood estimation. $\alpha =1$ corresponds to a heavily tailed distribution, whereas increasing $\alpha$ describes distributions with increasing evenness (Additional file 1: Fig. S1). Outlying samples were detected with Hubert’s criterion for skewed distributions [47]. For that, four different conditions were examined: (i) the number of unique TCR sequences, (ii) the median number of TCR sequences, (iii) the total number of TCR sequences, and (iv) the skewness of the distribution $\alpha$. A sample was marked as an outlier if it was outlying in at least two of the four above-described conditions.

### Data subsampling

To estimate the robustness of diversity indices under various sequencing coverage (equivalent to a total number of TCR sequences), data subsampling was performed in which a subset of the TCR sequences was chosen at random, where the total number of chosen TCR sequences was varied. Specifically, for every dataset, a downsampling of sequences was performed, starting with the smallest number of total sequences in a sample across all the samples from all studies (36 sequences in an individual sample from Ostmeyer_2020 dataset) up to the median number of total sequences in each dataset (maximum value, different for every dataset). The intermediate range of total counts was chosen as eight values equally distributed on a logarithmic scale between the minimum and maximum values. Additionally, due to the large measurement intervals in the upper part of the total count’s range, four equally distributed values of subsampling counts were added, resulting altogether in 14 different subsampling counts for each sample. For every value of total counts, the data was sampled at random 100 times.

#### Diversity measures

The experimental and simulated abundance data were used to calculate 12 diversity measures. The calculations were done using the R package *vegan* [48], except for the Gini coefficient calculated using the package *ineq *[49].

#### Richness

Richness is defined as a number of unique TCR sequences detected in an investigated repertoire. In this work, this index is denoted as *S*.

#### Chao1 index

The Chao1 index was introduced as an estimator of species richness in a population [50]. It estimates the impact of rare TCR sequences: singletons (${n}_{1}$—number of TCR sequences having 1 clone only) and doubletons (${n}_{2}$—number of TCR sequences having 2 clones only), and is defined as:

2 $${Chao}_{1}=S+ \frac{{n}_{1}({n}_{1}-1)}{2({n}_{2}+1)}$$

In this work, this index is denoted as *Chao1*.

#### ACE index

The ACE estimator is a nonparametric estimator of species richness in a population [51]. It estimates the impact of rare TCR sequences, where the threshold of rare sequences is on a default set to 10. It is defined as:

3 $$ACE={S}_{abund}+ \frac{{S}_{rare}}{{C}_{ace}}+\frac{{n}_{1}}{{C}_{ace}}{\gamma }_{ace}^{2}$$

where

4 $${C}_{ace}=1- \frac{{n}_{1}}{{N}_{rare}}$$

5 $${\gamma }_{ace}^{2}=\text{max}[ \frac{{S}_{rare}{\sum }_{i=1}^{10}i\left(i-1\right){n}_{i}}{{C}_{ace}{N}_{rare}\left({N}_{rare}-1\right)}-1, 0]$$

Here ${n}_{i}$ refers to the number of TCR sequences having $i$ number of clones, ${S}_{abund}$ corresponds to the number of unique TCR sequences with more than 10 copies, ${S}_{rare}$ corresponds to the number of unique TCR sequences of 1 to 10 copies, ${N}_{rare}$ is a total number of TCR sequences with 1 to 10 copies. In this work, this index is denoted as *ACE*.

#### D50

The D50 estimator is defined as the smallest percentage of unique, dominant TCR sequences that make up for at least 50% of the total TCR sequences in an analyzed population [52]. In this work, this index is denoted as *d50*.

#### Hill numbers

Hill numbers are exponentials of an information measure called Rényi entropy [19]. The exponential behavior of Rényi entropies can be counterintuitive, so the quantification of diversity via Hill numbers may be more easily understood [19]. The Hill number of order $q\ne 1$, ${D}_{q}$ is introduced in [19] as:

6 $${D}_{q}={\left({\sum }_{i=1}^{S}{p}_{i}^{q}\right)}^{1/(1-q)}$$

where $$S$$ is the richness of the sample and $${p}_{i}$$, $$i\in \{\text{1,2},\text{3,4},\cdots R\}$$ is the relative abundance of the *i*th clonotype or species. Hill numbers can also be expressed as the reciprocal of a generalized mean of relative abundances. In this sense, they can be understood as the effective number of species, with the continuous parameter $$q$$ controlling the emphasis placed on rare species. In this work, two Hill numbers (for $$q=3$$ and $$q=4)$$ were calculated and denoted as *D3* and *D4*, respectively.

#### Shannon entropy

${D}_{1}$ Obtained through substituting $q=1$ into equation $(6)$ is undefined. However, we can define ${D}_{1}$ as the limit of ${D}_{q}$, which is found to be [19]:

7 $${D}_{1}={e}^{-{\sum }_{i=1}^{S}{p}_{i}\text{ln}\ {p}_{i}}$$

The natural logarithm of the equation $(7)$ is the Shannon entropy, $H$: an important entropy measure in information theory and widely used to quantify diversity in the fields of ecology and immunology. It is therefore defined as:

8 $$H= -{\sum }_{i=1}^{S}{p}_{i}\text{ln}\ {p}_{i}$$

In this work, the index is denoted as *Shannon*.

#### Inverse Simpson index

The Hill number of order $q=2$ corresponds to another common diversity measure, which is interpreted as a probability of selecting with replacement two clones of the same TCR sequence (9). The inverse of ${D}_{2}$ is called the inverse Simpson index:

9 $$Inverse\ Simpson=\frac{1}{\sum_{i=1}^{S}{p}_{i}^{2}}$$

In this work, the inverse Simpson index is denoted as *Inv.Simpson*.

#### Gini-Simpson index

Another measure based on ${D}_{2}$ is the Gini-Simpson index, defined as:

10 $$Gini-Simpson=1- \sum\nolimits_{i=1}^{S}{p}_{i}^{2}$$

It is equal to the probability that two clones chosen at random belong to a different TCR clonotype [20]. It takes values from the [0–1] range, where a high score indicates high diversity. In this work, the Gini-Simpson index is denoted as *Gini.Simpson*.

#### Pielou index and Basharin entropy

Pielou index was introduced as a measure of the evenness of a sample [18]. It is defined as:

11 $$J=\frac{H }{\text{ln}\ S }$$

where *H* is Shannon entropy and *S* is the sample richness, which is equal to the maximum entropy. The score varies between 0 and 1, where a high value indicates an even distribution of TCR sequences. Worth noticing, in TCR analysis very often the complement of Pielou index $1-J$ is used to measure the clonality of the repertoire.

Basharin’s estimator of entropy is a modification of the Pielou index [18], which includes the adjustment of richness divided by the total number of all measured TCR sequences $N$ and is defined as:

12 $$J^{\prime}=\frac{H- \frac{S-1}{2N} }{\text{ln}\ S}$$

In this work, the Pielou index (11) is denoted as *Pielou*, whereas the adjusted Pielou index (12) is denoted as *Basharin*.

#### Gini coefficient

The Gini coefficient is a measure widely used to measure income inequality [15]. It is defined as:

13 $$Gini =\frac{{\sum }_{i=1}^{S}{\sum }_{j=1}^{S}\left|{p}_{i}-{p}_{j}\right|}{2{ S}^{2} \overline{p} }$$

where $\overline{p }$ is the mean relative abundance in the sample. It ranges between $0$ and 1 and a low value indicates a more even population. A Gini coefficient of $0$ means all clonotypes are equally abundant. In this work, the Gini index is denoted as *Gini.*

#### Grouping diversity indices

The indices were categorized into three groups based on their formulas and ability to explain richness, evenness, or both. *S*, *Chao1*, and *ACE* were grouped as indices describing only richness. *Shannon*,* Inv.Simpson*,* Gini.Simpson*,* D3*, and *D4* were grouped as indices explaining richness and evenness. *Pielou*, *Basharin*, *d50*, and *Gini* created a category of indices describing only evenness*.*

### Statistical testing

For the simulated data, the impact of varying *Evenness* or *Richness* on each diversity index was estimated using variable importance measures from three non-parametric models including random forest model (RF), generalized additive model (GAM), and multivariate adaptive regression spline (MARS) implemented in the *caret* R package [53]. The method to calculate variable importance is different for all algorithms. For RF, for each tree, the prediction accuracy on the out-of-bag portion of the data is recorded. Then the same is done after permuting each predictor variable. The difference between the two accuracies is then averaged over all trees and normalized by the standard error. For GAM, the model coefficients were used. For MARS, the changes in the generalized cross-validation estimate of error for each predictor are tracked and reductions in the statistic when each predictor’s feature is added to the model are accumulated. The variable importance is scaled to sum up to 100% per model.

For the real data, the correlation coefficient was calculated between every diversity index, considering all samples from all datasets together. Both Pearson correlation, measuring linear relationships between variables, and Spearman correlation, measuring monotonic association between the variables, were measured. Next, statistical testing for the case–control studies (Table 1) was performed, using the *U* Mann–Whitney test, and the corresponding effect size statistic was calculated as the rank-biserial coefficient of correlation (RBCC) estimated with Wendt’s formula [54]. RBCC ranges from 0 to 1, where increasing values correspond to increasing association between the groups analyzed. The thresholds to distinguish small, medium, high, and very high effects are as follows: 0.1, 0.3, 0.5, and 0.7 respectively. To estimate the robustness of indices depending on coverage (i.e., the total number of TCR sequences), coefficients of variation (CV), and the absolute value of relative error (RE) were calculated on subsampled data for every dataset. The closer CV and RE are to 0, the more robust the diversity index. Next, linear regression models were fitted to quantify the changes in CV and RE based on the total coverage indicated with subsampling. The closer the estimated slope coefficient to zero, the more stable the diversity index is (Additional file 1: Fig. S4, Additional file 1: Fig. S5). Using median CV and RE, as well as slope coefficients of models for CV and RE, a ranking of diversity indices was created, as the sum of ranks in all datasets. The smaller the sum of ranks, the better the overall performance of the index.

## Supplementary Information

Additional file 1: Figures S1-S5. Fig. S1—Visualization of simulated data diversity for selected values of Richness and Evenness. Fig. S2—Coefficients of variation of the diversity indices among Evenness for changing Richness valuesand among Richness for changing Evenness values. Fig. S3—Diversity indices calculated for subsampled data, with varying total number of TCR sequences. Fig. S4—Coefficients of variationfor D3 diversity index for subsampled sets of TCR sequences. Fig. S5—Linear regression models for D3 diversity index for subsampled sets of TCR sequences

## Data Availability

No datasets were generated or analysed during the current study.

## Abbreviations

BCR: B cell receptor
CV: Coefficient of variation
GAM: General additive model
MARS: Multivariate adaptive regression spline
TCR: T cell receptor
r: Pearson coefficient of correlation
R^2^: Coefficient of determination
RBCC: Rank biserial coefficient of correlation (effect size metric)
RE: Relative error
RF: Random forest model
