# Supplementary material for: A comprehensive evaluation of diversity measures for TCR repertoire profiling
Source: BMC Biol. 2025 May 14;23:133. doi: 10.1186/s12915-025-02236-5 (PMC12080070; doi:10.1186/s12915-025-02236-5)
Supplement: Supplementary file 1 — Additional file 1: Figures S1-S5. Fig. S1—Visualization of simulated data diversity for selected values of Richness and Evenness. Fig. S2—Coefficients of variation of the diversity indices among Evenness for changing Richness valuesand among Richness for changing Evenness values. Fig. S3—Diversity indices calculated for subsampled data, with varying total number of TCR sequences. Fig. S4—Coefficients of variationfor D3 diversity index for subsampled sets of TCR sequences. Fig. S5—Linear regression models for D3 diversity index for subsampled sets of TCR sequences [file 12915_2025_2236_MOESM1_ESM.docx]

**A comprehensive evaluation of diversity measures for TCR repertoire profiling**

Supplementary information


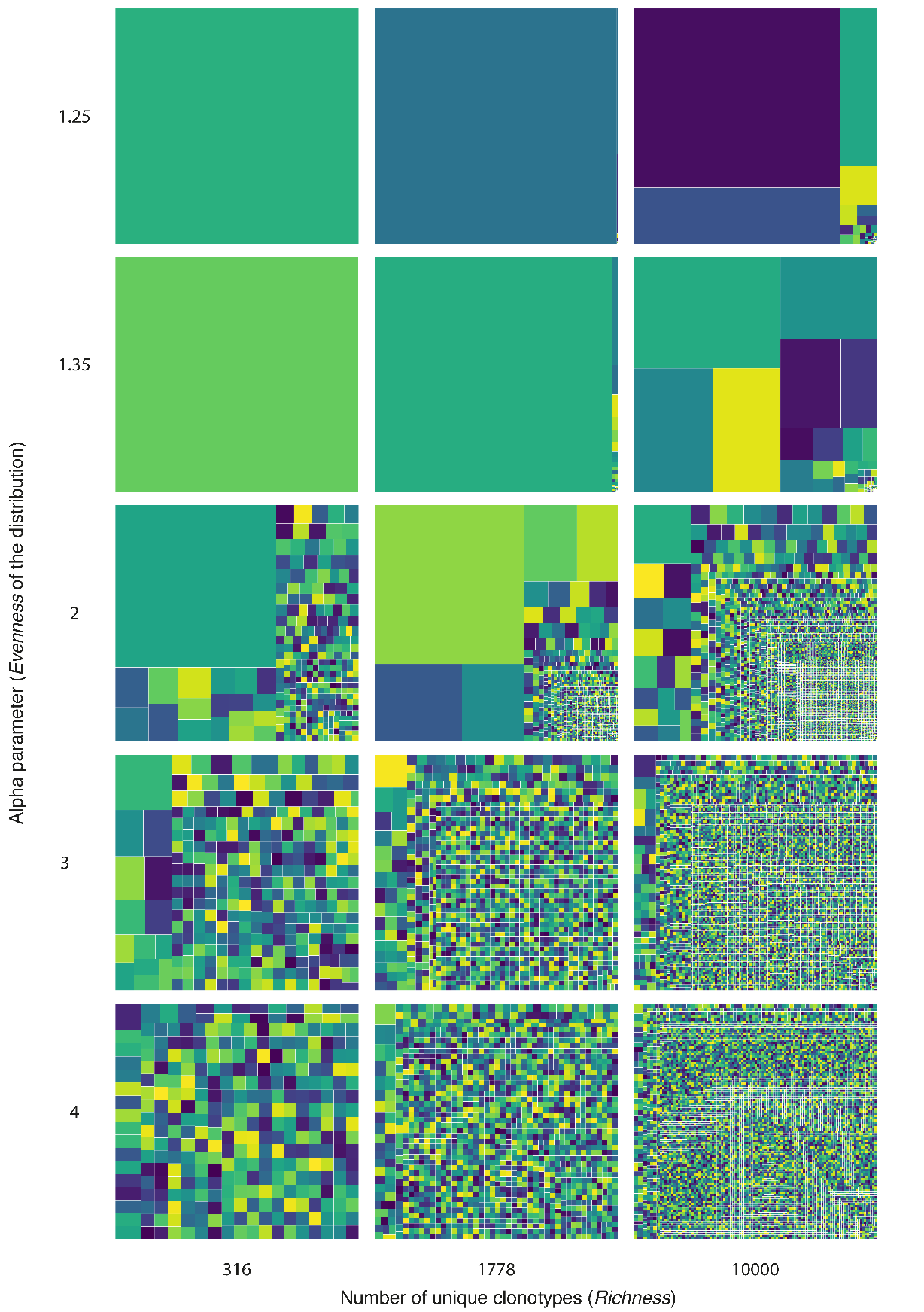


**Fig. S1. Visualization of simulated data diversity for selected values of Richness and Evenness.** The higher the alpha (Evenness), the more evenly distributed TCR clones. Richness corresponds to the number of unique TCR sequences.


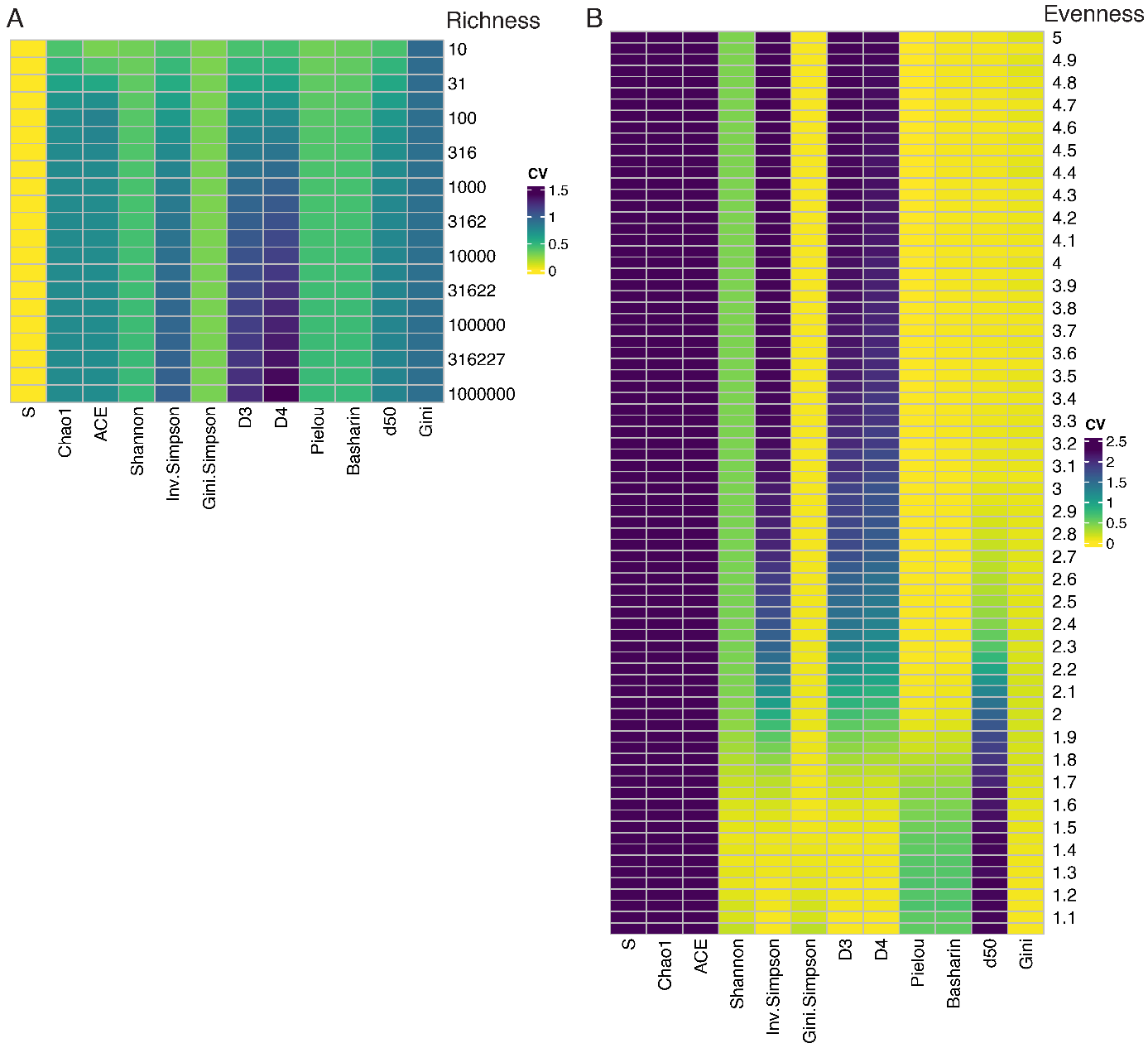


**Fig. S2. Coefficients of variation of the diversity indices among Evenness for changing Richness values (A) and among Richness for changing Evenness values (B).** Simulated data considered.


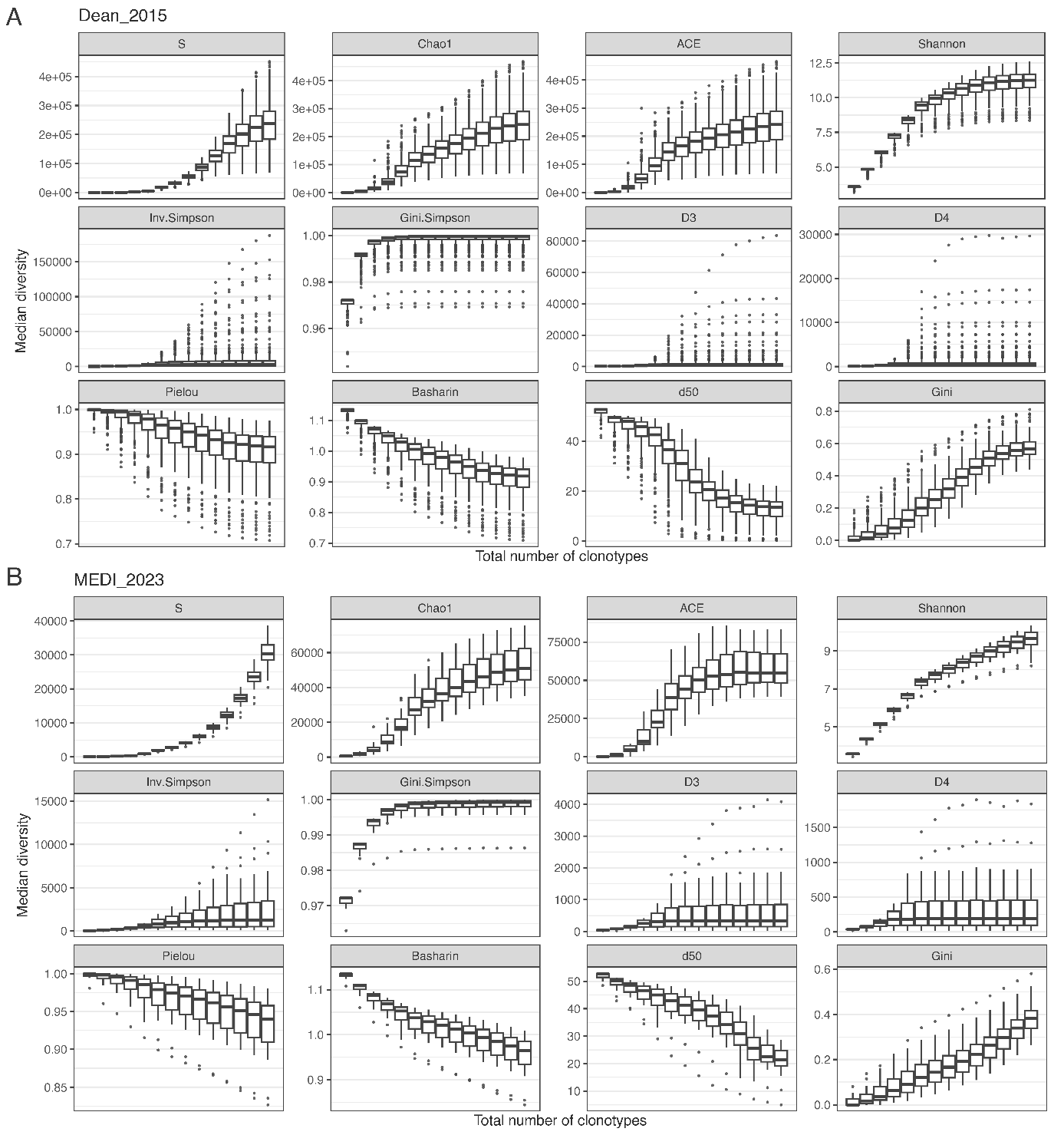


**Fig. S3. Diversity indices calculated for subsampled data, with varying total number of TCR sequences.** Boxplots show diversity calculated for 100 repeats of TCR sequences subsampling for two datasets represented by the skewed (A; Dean dataset) and uniform (B; MEDI dataset) distribution of sequences.


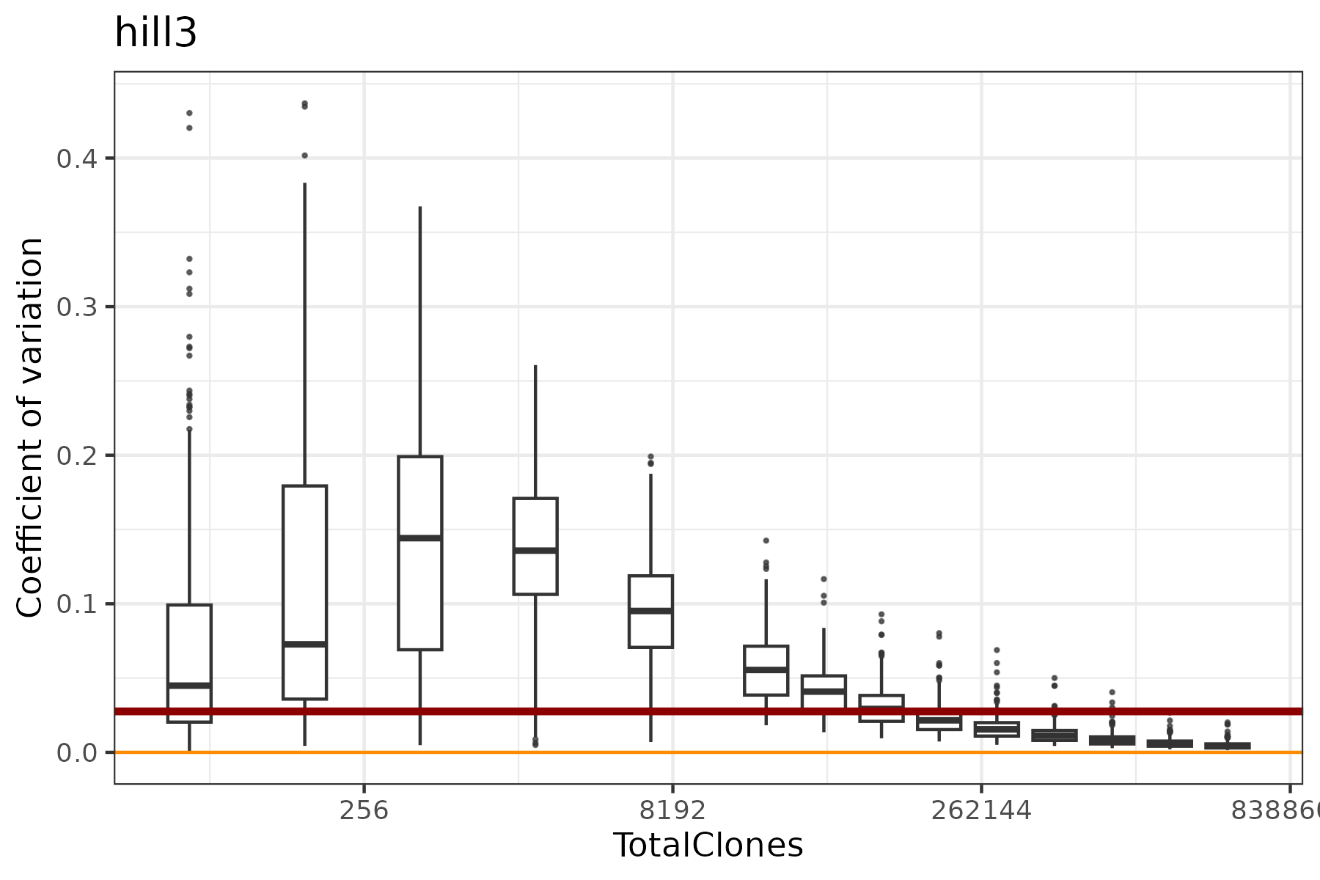


**Fig. S4. Coefficients of variation (CV) for D3 diversity index for subsampled sets of TCR sequences.** The baseline represents no variation using an orange color. The median value of CV calculated across all sequencing depths is colored with dark red. The closer the median CV to 0, the less variation of an index for all sequencing depth. The total number of TCR clones on the x-axis is presented in a logarithmic scale.


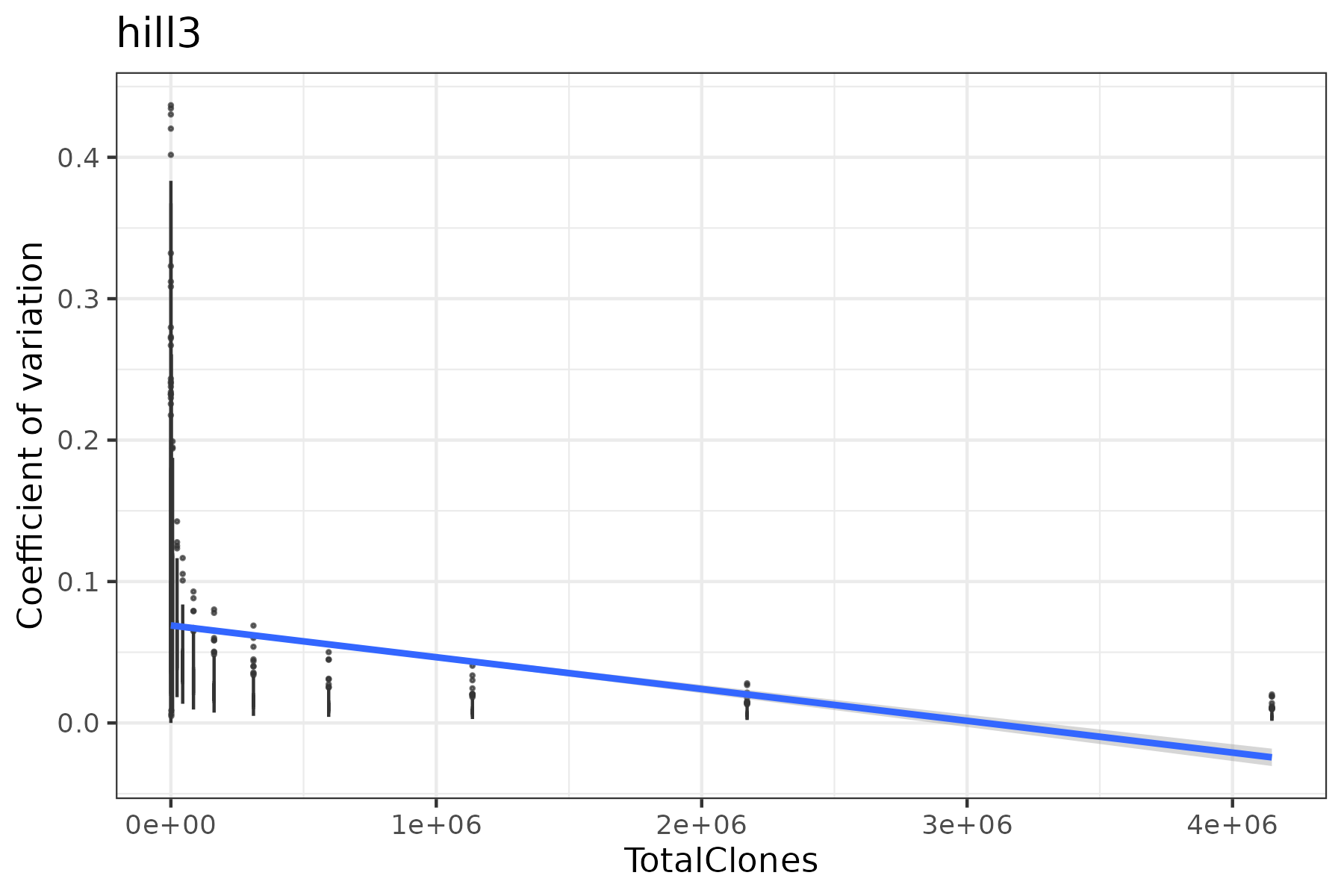


**Fig. S5. Linear regression models for D3 diversity index for subsampled sets of TCR sequences.** Linear models were constructed to investigate the impact of sequencing depth. The slope coefficient shows the direction of changes in CV/RE regarding the sequencing depth. The closer the slope to 0, the more stable the diversity index. The figure below shows boxplots of CV for different sequencing depths (x-axis in normal scale) with linear regression lines in blue.
